# Pediatric Kidney Transplantation—Can We Do Better? The Promise and Limitations of Epitope/Eplet Matching

**DOI:** 10.3389/fped.2022.893002

**Published:** 2022-06-03

**Authors:** Olga Charnaya, Daniella Levy Erez, Sandra Amaral, Dimitrios S. Monos

**Affiliations:** ^1^Department of Pediatrics, Johns Hopkins University School of Medicine, Baltimore, MD, United States; ^2^Schneider Children's Medical Center, Institute of Pediatric Nephrology, Petah Tikvah, Israel; ^3^Departments of Pediatric Nephrology and Biostatistics, Epidemiology and Informatics, Children's Hospital of Philadelphia and Perelman School of Medicine, University of Pennsylvania, Philadelphia, PA, United States; ^4^Department of Pathology and Laboratory Medicine, Children's Hospital of Philadelphia and Perelman School of Medicine, University of Pennsylvania, Philadelphia, PA, United States

**Keywords:** kidney transplant, epitope, eplet, pediatric, donor specific antibodies

## Abstract

Kidney transplant is the optimal treatment for end-stage kidney disease as it offers significant survival and quality of life advantages over dialysis. While recent advances have significantly improved early graft outcomes, long-term overall graft survival has remained largely unchanged for the last 20 years. Due to the young age at which children receive their first transplant, most children will require multiple transplants during their lifetime. Each subsequent transplant becomes more difficult because of the development of *de novo* donor specific HLA antibodies (dnDSA), thereby limiting the donor pool and increasing mortality and morbidity due to longer time on dialysis awaiting re-transplantation. Secondary prevention of dnDSA through increased post-transplant immunosuppression in children is constrained by a significant risk for viral and oncologic complications. There are currently no FDA-approved therapies that can meaningfully reduce dnDSA burden or improve long-term allograft outcomes. Therefore, primary prevention strategies aimed at reducing the risk of dnDSA formation would allow for the best possible long-term allograft outcomes without the adverse complications associated with over-immunosuppression. Epitope matching, which provides a more nuanced assessment of immunological compatibility between donor and recipient, offers the potential for improved donor selection. Although epitope matching is promising, it has not yet been readily applied in the clinical setting. Our review will describe current strengths and limitations of epitope matching software, the evidence for and against improved outcomes with epitope matching, discussion of eplet load vs. variable immunogenicity, and conclude with a discussion of the delicate balance of improving matching without disadvantaging certain populations.

## Key Points

- Epitope matching between donor and recipients provides additional benefit over antigen-level matching.
- Numerous software programs are available for comparison but require high-resolution tissue typing for accurate results.
- Enumeration of mismatch does not account for variable immunogenicity of epitopes.

## Introduction

Kidney transplant (KT) is the optimal treatment for end-stage kidney disease (ESKD) as it offers significant mortality and quality of life advantages over dialysis. While advances in immunosuppressive pharmacology and surgical techniques have significantly improved early graft outcomes, long-term graft survival has remained largely unchanged for the last 20 years. Due to the young age at which children receive their first transplant and additional 40–60 year life expectancy, children will require multiple transplants during their lifetime. Development of *de novo* donor specific antibodies (*dnDSA)* is associated with shortened graft survival making each subsequent transplant more difficult by limiting the donor pool and increasing mortality due to longer time on dialysis awaiting re-transplantation (1, 2).

Reducing the formation of *dnDSA* can be achieved through secondary prevention by increasing post-transplant immunosuppression. However, this approach is limited in children by a significantly increased risk for viral and oncologic complications (3–5). Additionally, there is evidence showing improvement in cardiovascular risk factors (blood pressure and lipids) and improved growth, with comparable rates of acute rejection and graft survival, for children on steroid-free and reduced intensity immunosuppression protocols (6–8). There are currently no FDA-approved therapies that have been shown to meaningfully reduce *dnDSA* burden or improve long-term allograft outcomes (9–11). Primary prevention strategies aimed at reducing the risk of *dnDSA* formation would allow for the best possible long-term allograft outcomes without the adverse complications from over-immunosuppression. One mechanism by which this can be achieved is by increasing precision of immunological matching at the time of transplant.

**Figure 1:**
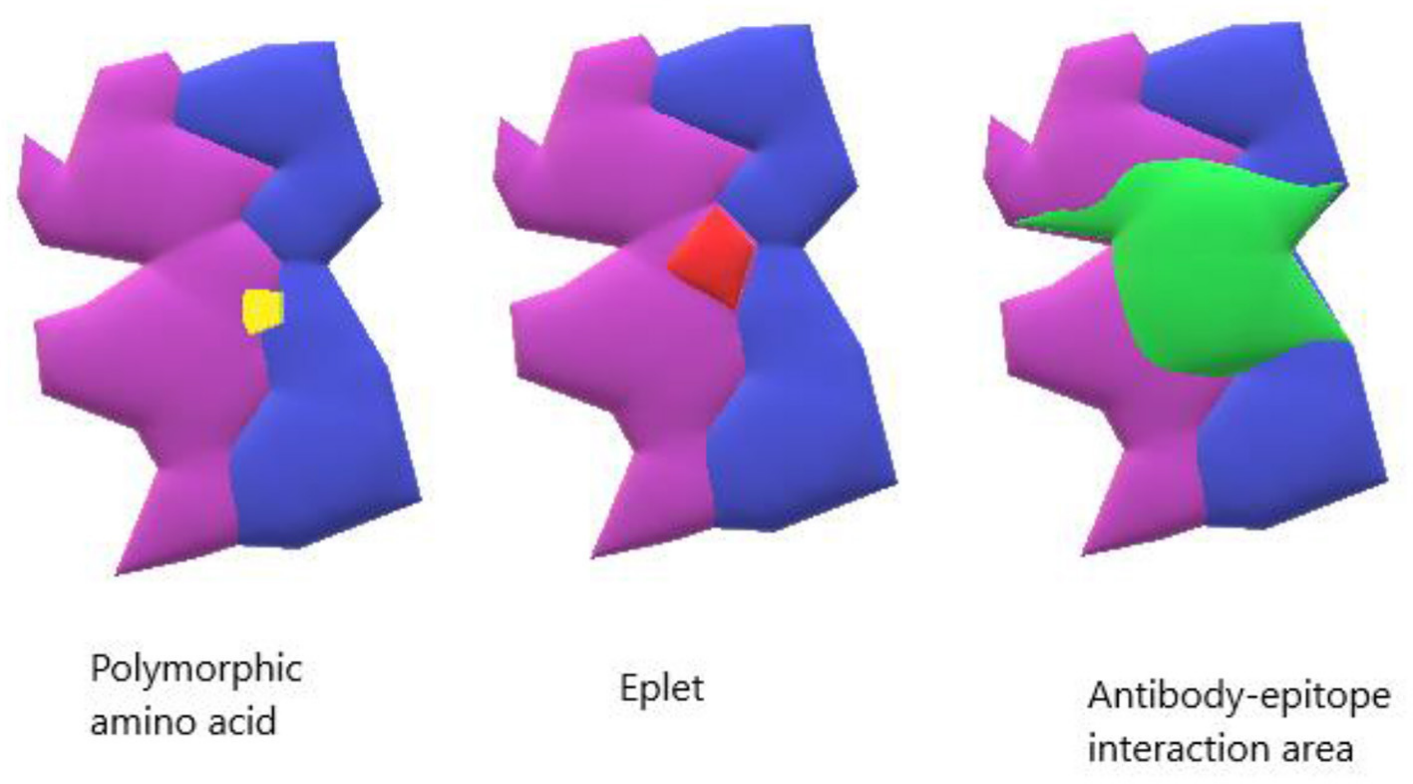
Model of HLA binding groove. The yellow shows a single polymorphic amino acid change. The red shows an antibody-verified eplet. The green shows the entire antibody interaction area which can contain the surface area for antibody-epitope interaction.

Traditional immunological matching has been done with a comparison of whole molecule HLA characterization at 3 loci (A, B, and DR). As the methods of accurate HLA characterization improved, it became clear that different HLA alleles had variable number of amino acid differences ranging from one to many, depending on the compared alleles. Simply enumerating HLA allele mismatches was insufficient to account for structural differences with immunological significance. Eventually, NGS (Next Generation Sequencing), a method that produces accurate genotyping at the molecular level, enabled improved characterization of HLA molecules by identifying the amino acid differences among multiple HLA alleles/molecules. The term “high-resolution tissue typing” or two-field (HR-2F) typing has been used to describe the level of characterization that enables identification of all 11 HLA antigens at the protein/molecular level, and thereby allows an epitope compatibility comparison between the donor and recipient (12). It should be clarified that the term “epitope” is used to describe any amino acid differences between two HLA molecules, whether recognizable by an antibody (Ab) or not, while the term “eplet” was coined by Rene Duquesnoy to indicate the structural elements (clusters of 2-5 polymorphic amino acids), linear or conformational on an HLA molecule recognized by an anti-HLA antibody (Figure 1) (13, 14). Comparison of eplets/epitopes, rather than whole HLA molecules, enables assessment of immunological compatibility with significantly more detail. Previous epitope/eplet mismatch (EMM) studies have reported an association of epitope/eplet load with allograft outcomes, rejection, and the formation of *dnDSA* (15, 16). Compared to low-resolution HLA matching, EMM predicted an incremental risk of HLA sensitization for increasing number of mismatches and offers additional value in predicting *dnDSA* formation (17, 18).

This review describes the different software tools currently available for comparison of high-resolution tissue typing, their predictive capability and clinical applicability, and discusses the nuances of variable immunogenicity. Practice applications of this technology, limitations and remaining knowledge gaps will also be also addressed.

### Strengths/Limitations of Available Software

To support informed clinical decision making, the comparison of donor and recipient HLA types relies on various software applications to identify immunological similarities and differences between pairs. While each has their own approach, the general principle employed by all is that a stronger recipient T/B-cell response is more likely to occur the more different a donor is from the recipient, and therefore the software generates a score to quantify the dissimilarity between the donor and recipient (19). There are currently three commonly internationally used software programs: HLAMatchmaker, single amino acid MM (AMS), and PIRCHE. Each program is described here with emphasis on advantages and disadvantages. The programs are compared in Table 1.

**Table 1:** Comparison of the three commonly utilized software packages utilized for comparison of donor and recipient HLA types in solid organ transplantation.

	**Analysis**	**Results**	**Limitations**
HLAMatchmaker	Comparison of continuous and discontinuous eplets.	Eplet load	Software will categorize mismatched eplets as “verified” or “unverified”, however this can be misleading as many were not tested. Treats both recipient and donor alleles as single entities.
Amino acid sequence comparison	Direct comparison of the number of mismatches between donor and recipient HLA allele amino acid sequences.	Amino acid mismatch score	Considers all mismatches equally important. Standard method doesn't account for physicochemical parameters.
PIRCHE	Prediction of which donor-derived peptides can be presented to T-cells in the context of recipient HLA-DR molecule.	PIRCHE score	The algorithm does not consider mismatched epitopes presented by HLA-DQ, DP and DR3/4/5.

### HLAMatchmaker

HLAMatchmaker, the first developed and most common software, was developed by Rene Duquesnoy in 2002 (20). It is a structurally based matching program based on the understanding that only a portion of the HLA antigen is recognized by HLA antibodies. HLAMatchmaker uses donor/recipient amino acid sequences to determine continuous and discontinuous eplets that are likely part of the area recognized by an antibody's complementary-determining region. HLAMatchmaker focuses only on polymorphic regions and provides information regarding “verified” and “non-verified” by antibody reactivity epitopes. All mismatched eplets are assigned the same value for a sum of eplet mismatch load (19, 21). The number of eplets that are mismatched between donor and recipients are calculated by the software either at each HLA locus by class (I or II), or as a cumulative number. The HLAMatchmaker software has been well validated and has been shown to predict both *dnDSA* formation, rejection, and graft failure rates (20, 22–24). Given the nature of the software, the “non-verified” epitopes represent mismatched elements that may potentially produce antibodies but have not been proven to do so; therefore, the need for the broader term “epitope” which does not need Ab reactivity verification. Thus, two donor/recipient pairs may have similar absolute numbers of epitope mismatches, but may vary substantially in their impact on antibody production (21). In addition, the *HLAMatchmaker* site must be continually updated as more epitopes become antibody verified. The last update of the program at the time of this publication was July 2020.

### Amino Acid Sequence Comparison

The simplest method is comparison of donor and recipient amino acid sequences, enumerating the number of mismatches generating an amino acid mismatch score (AMS). Akin to eplet mismatch in the HLAMatchmaker program, this method assigns an equal weight to all mismatches but does not make any assumptions about which areas of the molecule may be more relevant for antibody recognition (19). In multivariable models, the odds ratio for developing DSA increased for every 10 amino acid mismatches, with a larger effect size for Class II than Class I DSA, however both were statistically significant (17).

The assessment of various physicochemical parameters may augment the AMS metric, and there have been numerous approaches including Grantham's distance or Epstein coefficient of difference (25, 26). These measures are calculations of amino acid (aa) properties that seek to provide a quantitative measure of the physicochemical change associated with specific aa substitutions in proteins. Even though these are rather simplified models, they are most likely more informative than simple aa mismatch enumeration. More specifically assessing protein to protein differences using physicochemical characteristics have been adopted with some degree of success showing some benefit over simple enumeration (13, 27, 28).

There remains much potential and promising work in methods that seek to incorporate three-dimensional (3D) contextual information into MM scoring systems (29, 30).

### Predicted Indirectly Recognizable HLA Epitopes (PIRCHE)

The complex immune alloreactivity after solid organ transplantations involves both T and B cells. Indirect T cell activation is proposed as a key player in the humoral response leading to dnDSA formation (31). The PIRCHE algorithm identifies which mismatched HLA epitopes can be recognized by T cells needed to activate the B-cell response. The output from the software includes a score which predicts the risk of DSA formation. Studies have found good correlation between PIRCHE score, DSA formation, and allograft outcome (32–34).

A recent comparative study among KT recipients EMM, AMS, and AMS as augmented by electrostatic mismatch showed that each method was independently associated with DSA formation after adjustment for recipient age, immunosuppression regiment, and non-adherence without evidence of superiority of either approach (35). Meneghini et al. compared the 3 methods of analysis among a cohort of 169 KT recipients and found a strong correlation between the three software scores, however only the PIRCHE score was found to be an independent predictor of donor-specific T-cell response in multivariable analysis, as would be expected (36). Similarly, in a study of 2,787 transplants PIRCHE-II and HLAMatchmaker were both found to independently predict DSA formation in multivariable analysis (controlling for the other method) suggesting that these approaches may complement each other (37). Combining the software approaches and understanding the immune alloreactivity mechanism where both T and B cells are involved, will likely be the most informative approach. Efforts to combine them have already been initiated and presented at the most recent meeting of America Society of Histocompatibility and Immunogenetics (38).

It is important to understand that despite the improved computational analysis software there are limitations. The needed HR-2F typing is available in living donation but not in deceased donors. The NGS technologies until recently did not allow a turn-around time of <7 h for HR-2F, however recent advances have taken a large leap towards ameliorating this limitation (39, 40). To address this problem our community has attempted to impute HR-2F HLA typing utilizing low or even intermediate level typing and an NMDP database called HaploStats or even HLAMatchmaker. Engen et al., studied the accuracy of imputation techniques and showed that only 35.6% of the imputed haplotypes had no mistakes, and HLAMatchmaker was not able to provide high-resolution haplotypes for 45.2% of Caucasian subjects and 63.5% of non-Caucasian subjects (*p* = 0.002) (41). This study reinforces that bypassing direct and accurate characterization of HLAs at the HR-2F introduces significant bias into the analysis by currently available computational software.

## Epitope Matching Impact on Clinical Outcomes

Epitope/eplet based matching has been suggested as a more precise strategy to improve immunologic outcomes with reduced formation of *dn*DSA compared to antigen-level matching (13, 42–46). It is known that chronic antibody-mediated rejection (ABMR), due to *dn*DSA is the major cause of late graft loss (15). DSA directed against HLA antigens are central to the development of ABMR. It was also found that formation of *dn*DSA vary and depend on the number and type of amino acid mismatches between donor and recipient, the electrostatic effect of these changes and the conformational differences in epitopes (47). The important question is what evidence exists correlating using epitope matching to improve clinical outcomes, specifically reducing rejection rates and graft loss.

Philogene et al. demonstrated in a cross-sectional study of 110 racially diverse pediatric KT recipients that an HLA class I eplet load >70 resulted in a greater risk of rejection compared to same race recipient and donor (48). A second retrospective analysis of 151 living donor KT recipients in a European cohort evaluated incidence of ABMR in patients transplanted with low, moderate and high HLA-II epitope mismatch load. ABMR incidence rates were 2, 14, and 13%, respectively, at 96 months (*p* = 0.036). A sub-analysis including only patients with no preformed DSA (*n* = 138) demonstrated similar results (49).

Sapir-Pichhadze et al. studied a large American cohort of 118,382 first KT recipients and assessed death censored graft failure based on antibody verified EMM. Antibody-verified eplet mismatches were found to be independent predictors of death-censored graft failure (50). Their team validated the results in an independent Canadian cohort of 52 patients with transplant glomerulopathy and 104 controls, with similar findings. Collectively, these results support donor-recipient matching for specific HLA eplets to reduce the risk of graft loss.

In adult kidney transplantation there is a growing body of literature showing increased risk for DSA formation, ABMR, and allograft failure with increasing eplet/epitope mismatch score (13, 16, 51, 52). In children, there is less published literature, however, most research implementing EMM in pediatric KT has focused on eplet load, an enumeration of eplets mismatched between the donor and recipient (48, 53, 54). Eplet load has been linked to adverse allograft outcomes including acute rejection, allograft loss, and transplant glomerulopathy (50, 51, 55).

## Load vs. Immunogenicity

Despite the ample evidence for superior ability to risk stratify and predict adverse outcomes by optimizing matching with eplet load testing, perfect immunological matching at the allele or eplet level is impractical. Over-prioritization of HLA matching results in prolonged waitlist time and must be balanced with the morbidity and mortality of advanced CKD and dialysis including increased risk for cardiovascular disease (56, 57). The prolonged waitlist time is unequally burdensome and disadvantages patients from racial and ethnic minority populations who express rare HLA genotypes (58, 59).

Eplet load does not account for the electrostatic and conformational characteristics that result in variable antigen immunogenicity (17, 60). The concept that some eplets are more immunogenic than others was explored in a retrospective study in heart and lung transplant recipients; specific eplet mismatches were found to be disproportionally associated with *dn*DSA formation and modeling avoidance of these high-risk eplets predicted a significant reduction in *dn*DSA formation (43). Using imputation to determine HR-2F HLA type in the Scientific Registry of Transplant Recipients (SRTR), a subset of 15 eplet mismatches was linked to a higher hazard of death-censored graft failure among KT recipients, even after accounting for other known risk factors (61, 62). Additionally, a small subset of Ab-verified eplets have been linked to transplant glomerulopathy in an independent Canadian KT cohort (50). These innovative studies are laying the foundation for incorporating variable immunogenicity into the decision framework for donor allocation and acceptance. One major limitation of these studies is the use of imputation to determine HLA type, which is prone to error especially in racial and ethnic minorities (63). The authors appropriately identified this limitation and used sensitivity analysis to compare findings in Caucasian patients with those of other races with similar results.

Just as not all eplets are equally immunogenic, not all DSA are equally pathogenic and as such, eplet load is insufficient to inform risk stratification for DSA most likely to lead to adverse allograft outcomes (13, 16, 64, 65). A novel approach to study the variable immunogenicity of HLA-DQ eplets was designed by Schawalder et al., utilizing pregnancy as the immunological trigger, allowing a study of variable immunogenicity without the need to account for medication non-adherence and other significant post-transplant complications (66). The team identified several DQB1 and DQA1 eplets with higher immunogenicity scores in a primarily Caucasian study cohort, however these findings still need to be confirmed in a broader, more ethnically diverse cohort and then applied to a robust transplant cohort to examine the immunomodulatory impact of immunosuppression on the host response. The Schawalder study also highlighted the challenges of studying variable immunogenicity as the immune response is often complex and assigning antibody specificity to a specific eplet when multiple mismatched eplets on the same HLA molecule are present is fraught with bias. Tambur et al. studied a cohort of KT recipients with 2 DQ mismatches where *dnDSA* was generated to only 1 of the DQ antigens (*2MM1DSA cohort*) and showed significant inconsistency with eplet load, affirming that EMM is a significant correlate of rejection at the population level however is not sufficiently informative to serve as a predictor at the individual level (64). A recently described contributor to immunogenicity is the degree of expression of each HLA antigen, adding another level of complexity in assessing immunological risk. In a study of 103 pediatric solid organ transplant donor-recipient pairs, Shieh et al. showed DPB1 expression level was independently associated with DSA formation and EMM lost statistical significance in a multivariable regression model (67).

## Implementation

Implementation of EMM analysis has the potential to greatly improve the precision with which donors and recipients are matched, which can be especially beneficial for highly sensitized patients (68, 69). The principle hurdle to implementation of EMM analysis in deceased donor kidney allocation is the time necessary to perform high resolution tissue typing.

Until recently, most HLA laboratories used probe-based methods including sequence specific primer (SSP) and sequence-specific oligonucleotide prove typing (SSO) which generates low-intermediate resolution typing at the antigen level, and unable to provide the necessary allele-level specificity to allow EMM analysis. Exact genomic sequencing either through Sanger sequence-based typing (SBT) or Next-Generation sequencing (NGS), is gradually becoming the prevalent method for typing as laboratories seek to transition to a single assay for solid organ and bone marrow transplant programs. The major limitation to NGS is the 2–3 days needed for the assay and associated analysis.

In living donor KT, where the time to perform high-resolution typing is not a barrier, there are great possibilities for the application of this technology. The Australian Paired Kidney Exchange employs high-resolution typing and EMM score, utilizing this as a tool to aid in donor selection in addition to other pertinent clinical factors, and has described excellent outcomes in pediatric patients (70). The National Kidney Registry in the United States has started collecting data on EMM between donors and recipients, although no publication is yet available to compare the impact of adding EMM into donor selection considerations.

While the time element remains the main barrier to implementation of this methodology in deceased donor allocation on a large scale, several pilot and small-scale studies have shown benefit for pediatric KT recipients. Starting in January 2014, all patients listed for DDKT in Australia had SSO typing with imputation of alleles entered into the allocation program, and for each patient eplet load thresholds as well as unacceptable matches were pre-determined at the time of listing (53). Utilizing this method, children received DDKT with lower eplet loads compared to the previous allocation scheme, having an exceptionally significant impact on decreasing Class II load [Class II EMM 20.4 (5.4) vs. 63.7 (16.2), *p* < 0.001], however the study was limited by very small sample size. A pediatric center reported their practice of incorporating Class II EMM prospectively in 19 pediatric patients and showed that the adult reported DR and DQ eplet load thresholds may not be the same for pediatric patients. The paper reported only short-term outcomes and further data is needed to assess the impact on DSA formation and allograft survival (71). Recently, a novel nanopore sequencing technology was reported that can produce reliable, cost-effective, and rapid (<6 h) HR-2F typing (39, 40). While not yet ready for full-time clinical use, the ongoing development of such technologies will enable incorporation of EMM analysis into deceased donor kidney allocation.

Despite the promise of better immunological matching with eplet-based matching, we must be cognizant for the potential to worsen racial and ethnic disparities with over-prioritization of HLA matching. Prior to 2014, HLA matching was prioritized in deceased donor allocation in order to optimize long-term allograft survival. However, as non-White patients express rare HLA types, the likelihood of finding an HLA-matched donor for racial and ethnic minority patients is significantly reduced (58, 59). The 2014 Kidney Allocation System (KAS) deprioritized HLA matching and has narrowed the disparity in waitlist time among Black and Hispanic candidates and improved their transplant rates (72, 73). Unfortunately, the same policy had the unintended consequence of prolonging pediatric waitlist times, highlighting the need for critical appraisal that policy change can have on clinical outcomes (74–76). Because organ allocation is an entangled dynamic system, in which change to one factor can significantly impact the others, prioritization of only one element often fails and highlights the pressing need to develop an allocation model that can optimize immunological matching but that can preserve pediatric priority, as well as minimize racial and geographic disparities (77, 78).

In the United States, the Organ Procurement and Transplantation Network (OPTN) is reviewing allocation policy and strategies to improve equity with a proposal to create a composite allocation score in which candidates would get a summary score of different variables associated with their predicted need and potential for survival benefit, including factors such as age (pediatric priority), geographic proximity to donor, and sensitization. These models are under development and will require close monitoring after implementation. More data are needed to inform approaches to optimize matching for children and one approach might include a focus on class II matching (79).

## Discussion/Conclusion

Epitope/eplet matching holds the promise of better immunological compatibility between donors and recipients thereby reducing risk for DSA formation. Despite many advances, many questions remain including the specific mechanisms of variable immunogenicity, technological barriers to rapid high-resolution molecular tissue typing, and balancing equity in an allocation system. The community however, is eager to use this approach, simply because molecular characterization is more meaningful than an allele name. Nevertheless, there remains considerable ambiguity in how to balance epitope mismatch with clinical factors including medical urgency (dialysis duration or limited access), age, psychosocial factors influencing adherence, and viral infection risk. It is very likely that the appropriate science and tools will soon be available for utilization and application, but additional studies are needed to identify the most efficient and effective approach to optimize outcomes.

## Author Contributions

OC developed the conceptual overview for the review. OC and DLE wrote the review and participated in critical review and revisions. SA and DM provided critical review and guidance. All authors approved the final version of the manuscript.

## Funding

This work was supported by (grant number KL2TR003099 to OC) from Johns Hopkins Institute for Clinical and Translational Research (ICTR).

## Conflict of Interest

DM is a consultant to and owns options in Omixon. DM receive royalties from Omixon. The remaining authors declare that the research was conducted in the absence of any commercial or financial relationships that could be construed as a potential conflict of interest.

## Publisher's Note

All claims expressed in this article are solely those of the authors and do not necessarily represent those of their affiliated organizations, or those of the publisher, the editors and the reviewers. Any product that may be evaluated in this article, or claim that may be made by its manufacturer, is not guaranteed or endorsed by the publisher.
